# *QuickStats*: Age-Adjusted Lung Cancer Death* Rates,^†^ by State — National Vital Statistics System, United States, 2018

**DOI:** 10.15585/mmwr.mm6936a8

**Published:** 2020-09-11

**Figure:**
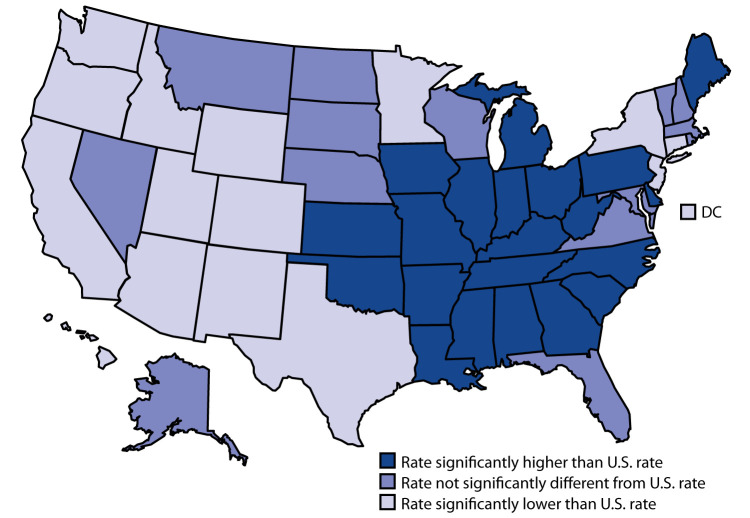
In 2018, the age-adjusted lung cancer death rate in the United States was 34.8 per 100,000. Twenty-one states had a higher lung cancer death rate than the national rate, 15 states and DC had lower death rates, and 14 states had rates that were not statistically different from the national rate. Most states with higher death rates were in the Midwest or Southeast. The five states with the highest age-adjusted lung cancer death rates were Kentucky (53.5), West Virginia (50.8), Mississippi (49.6), Arkansas (47.4), and Oklahoma (46.8). The five jurisdictions with the lowest lung cancer death rates were Utah (16.4), New Mexico (22.5), Colorado (23.0), DC (24.6), and California (25.0).

**Sources:** National Center for Health Statistics. National Vital Statistics System, mortality data. https://www.cdc.gov/nchs/nvss/deaths.htm; CDC. CDC WONDER online database. https://wonder.cdc.gov/ucd-icd10.html.

